# Exposing gendered microaggressions: the experiences of female physiotherapists working in men's professional sport

**DOI:** 10.3389/fspor.2026.1907581

**Published:** 2026-08-21

**Authors:** Melissa Reynolds, Heather Talberg, Fraser Carson

**Affiliations:** 1 Department of Health and Rehabilitation Sciences, Faculty of Health Sciences, University of Cape Town Anzio Road, Observatory, Cape Town, South Africa; 2 Department of Sport, Lunex University of Applied Sciences, Differdange, Luxembourg

**Keywords:** gender equity, gender inequality, professional practice, rugby union, workplace environment

## Abstract

**Objectives:**

The women's professional sports industry is experiencing rapid growth, and participation rates are higher than ever. This is encouraging more women to explore work opportunities in the industry. However, the sport workforce has a long-established hegemonic masculinity and many accepted behaviors and actions in male sport teams can be viewed as misogynistic. Despite initiatives to encourage more diversity, these behaviors can make it difficult for women to enter and challenging to remain working in men's professional sport.

**Methods:**

To explore the lived experience of women working in men's professional rugby union, a qualitative phenomenological research design was employed, using semi structured interviews, with 12 female physiotherapists. The interviews focused on the challenges they faced, and strategies used for navigating traditional masculinities and stereotypes

**Results:**

Thematic analysis identified four higher-order themes (1. Establishing professional identity in a male-dominated environment, 2. Navigating sexual objectification, 3. Career progression and organizational barriers, and 4. Work-life balance and personal costs) that depicted their experiences

**Discussion:**

Notwithstanding legislative changes to make sport more inclusive, these physiotherapists described persistent inequalities, microaggressions and exclusions. The findings highlight the need for structural and cultural reform in sport organizations to remove gender biases, and create professional environments where women's expertise is recognized, respected, and equitably supported.

## 1 Introduction^1^

Despite considerable advances in sport towards gender equity for athletes and other global figures indicating that women make up almost half of the participants at various levels of sport, there continues to be an underrepresentation of women in the broader sports workforce (1). Women continue to experience disproportionate career opportunities and advancement, reflected in disparities in hiring, pay, representation and treatment compared to men. Manifestations of this inequality include gendered stereotypes, sexual harassment, discriminatory policies, and exclusion from leadership roles. While female athletes have gained greater visibility, women working within the sports industry continue to face discrimination and structural disadvantage (2), especially when they hold some power within their position (3). As such, Sveinson et al. (4) called for more research to focus on data gathering around the lived experience of women and how everyday organizational practices marginalize them. Therefore, this study investigated the lived experience of women in the wider sport workforce to provide greater insights into the challenges faced and strategies that have been used to overcome these, helping to improve gender equity (5).

Hegemonic masculinity and traditional gender norms continue to perpetuate a male-dominated culture that marginalizes and overlooks women in various roles within the sports workforce (6). Rugby union is no exception, as rugby structures, as well as playing requirements, perpetuate masculine norms, where women encounter gendered barriers to participation, leadership and employment within organizations. Rugby is traditionally perceived as a sport embodying a typical form of dominant masculinity due to the nature of play favoring toughness and aggression. Within the team structure, masculine identities are reproduced along these embodied lines (7). Despite well-intentioned progress by World Rugby at a policy level to counteract gender inequalities, female rugby players face gendered marginalization, seen to be both invading male-coded spaces as well as violating feminine physicality norms (8). Given that gender inequity and bias are widespread in society generally, it can be assumed that this would also be present in a male-dominated environment.

Gender roles refer to the societal norms, behaviors, and expectations that dictate how individuals should act, speak, dress, and conduct themselves based on their assigned gender (9). Traditional gender roles have historically expected men to have an established career and be the breadwinner, with women taking on the role of primary caregiver. Women have been expected to be nurturing and gentle, while men are expected to be assertive and powerful (10). Many organizations have made improvements to create a more inclusive work environment, but more effort is required. For women in high-performance sport, starting a family is not accommodated and is a common reason for dropout (11), with women often unsupported managing dual roles of home and work. Marks et al. (12) noted that for women in sports academia, that career breaks from having children limited opportunities, inhibit potential to work unstructured hours and can influence job performance due to exhaustion.

Extensive research on coaching and sports leadership has consistently highlighted the underrepresentation of women in the sport workforce (1, 13, 14). In 2022, men accounted for 57% of the sports employment workforce across the European Union (EU), and in the United Kingdom (UK), 62% of sport-related roles were occupied by men (15, 16). In Australia, Dwyer et al. (17) estimated that only 24% of the high-performance sport workforce were women. Such figures point to persistent organizational bias, limiting women's opportunities for career progression (18). Despite regional differences, physiotherapy remains a female-orientated profession, with women representing 63% of the global workforce (19). Yet, the profession is split along gender lines with male physiotherapists disproportionately represented in sports and occupational health settings, while women are more frequently employed in hospitals, health promotion and rehabilitation settings (20). Men are in fact three times more likely to have chosen to pursue a career in physiotherapy due to an interest in sport (20). This gendered division highlights how traditional gender roles and constructs influence professional norms and opportunities within the field (21).

Sunde et al. (22) identified four recurring themes in experiences by women in the sports workforce: perceived ability, lack of respect, perseverance through negative experiences, and negative experiences leading to dropout. Many women reported the need to continually prove their competence, suppress feminine traits, and conform to masculine workplace norms (23). Sexism and gender-based comments remain common, often going unchallenged due to the absence of psychological safety (24). For female physiotherapists who manage to gain employment in high-performance sport, they often perceive a need to ignore or play down misogyny to keep their role and not be viewed as a troublemaker (25). These dynamics are sustained by hegemonic masculinity, entrenched societal stereotypes and limited organizational support for female staff (12).

There is limited research on the experiences of women working in traditionally male-dominated sports workforce domains. This includes physiotherapists working in professional men's sports and the unique challenges and barriers they may face. Conducting this research and gaining insights into their experiences may inform efforts to promote gender equality, challenge discriminatory practices, and create more inclusive environments within the sports industry (26). In this way, progress can be made towards dismantling barriers and striving for equal opportunities for all female professionals across the sports workforce. Facilitating the involvement of more women in the sports workforce would play a vital role in establishing sustainable sporting communities. As such, the purpose of this study was to explore the experience of female physiotherapists working in professional men's rugby union, focusing on the challenges they face within their role.

## 2 Methods

### 2.1 Study design

A qualitative social phenomenological research design (27), using semi-structured interviews, was employed to explore the lived experiences of female physiotherapists working in professional men's rugby union. This approach allowed for the individual's own interpretation of their environment to be central to their experience (28). Key to social phenomenology is the need to maintain a clear conceptual framework, be grounded in subjective meaning, keep the participants' viewpoint, and maintain consistency between the participants' responses and the research (29). The study was underpinned by an interpretivist paradigm, which views reality as socially constructed and emphasized understanding of how individuals make sense of their experiences in context (30). This approach aligned with the study aims and design, as it enabled participants to share their unique challenges and perspectives in their own words.

### 2.2 Participants

Semi-structured interviews were completed with 12 physiotherapists who were either currently employed by or had previously been employed by professional men's rugby union teams at either a national team or premier level. Participants were drawn from professional unions in both the Southern and Northern Hemisphere. This aligned with the primary researcher's own affiliations as a South African physiotherapist working in the field of sports physiotherapy in the United Kingdom. While recruitment initially targeted physiotherapists only from these two settings, slowness in recruiting resulted in the search for participants being expanded to other global contexts from teams who, despite being situated in different hemispheres, participate in shared competitions at a premier league level. The final pool represented four different nationalities, with seven of the 12 working with Northern Hemisphere clubs. Participants were all aged over 25 years (M = 34.5, SD = 7) and had been working in professional men's rugby union for an average of 5.3 years (Table 1). No specific demographic details linked to the club or franchises were captured to maintain the anonymity of the participants.

**Table 1:** Participant demographics.

Participant	Age	Employment location	Years of experience in professional sport	Years of experience in professional men's rugby union
1	45	SH	9	9
2	40	NH	12	12
3	27	NH	4	3
4	29	NH	7	1.5
5	38	SH	14	10
6	33	NH	7.5	4.5
7	28	NH	1.5	1.5
8	25	NH	1	1
9	33	NH	6	4
10	34	NH	7	5.5
11	47	SH	9	9
12	36	SH	6	3

NH, Northern Hemisphere; SH, Southern Hemisphere.

### 2.3 Procedure

Following institutional ethical approval (HREC Ref: 199/2023), a purposive criterion was used to recruit female physiotherapists currently or previously employed in professional men's rugby union (31). Snowball sampling further supported recruitment through participant referrals (32). Semi-structured interviews, guided by existing literature (2, 14, 33, 34), explored participants' experiences, challenges, and strategies for navigating traditional masculinities and stereotypes within rugby union. Examples of the questions include: What did the interview process look like when you applied for your role?; Can you tell about the dynamics of your relationship with the athletes and your fellow staff members?; Can you describe your interactions with rugby union management/organization?; What are the comments you hear most often when you tell someone that you are a physiotherapist in professional rugby union?; Do you feel you have the same job opportunities and career development opportunities as other physiotherapists with your experience? The flexible interview format ensured consistency while allowing unanticipated topics to emerge (35). Interviews were conducted by the primary researcher, online in English, lasting approximately 60 min, and were transcribed verbatim. Transcripts were returned to participants for member checking. Throughout the process, bracketing (29) was employed to acknowledge and minimize potential bias and preconceptions, with the primary researcher keeping a reflective journal and annotated memos to better understand and interpret the experiences and perspectives of the study participants, without undue influence from the researcher's own viewpoints. This reduced the unconscious bias that may have arisen from her position as a female physiotherapist working in a sports-focused setting. It is also accepted that participants may have been more receptive to sharing their experiences more freely with another female physiotherapist, providing insights that they may not have disclosed to someone outside their field or gender. All members of the research team used a reflexive approach when interpreting the results by consistently reflecting on the data through the analysis process and engaging in constructive discussion when differences in interpretation occur.

### 2.4 Data analysis

Analysis followed an inductive approach with initial codes and themes generated directly from the data to ensure that participants' lived experiences guided the early stages of interpretation. In the later stages, a deductive component was incorporated to contextualize and interpret the findings through LaVoi & Dutove's (2) socioecological model, which conceptualizes the multilevel, overlapping barriers and supports affecting women's participation and retention in the sport workforce. At the most proximal level, the model includes “personal, biological, and psychological factors such as cognition, emotions, beliefs, values, expertise, and personality of the individual” (36) p. 57; i.e., low self-confidence). The second interpersonal level reflects the influence of relationships and networks, which can provide support and mentorship or reinforce barriers through bias and exclusion (i.e., family relations). Moving outward, the organizational level captures structural and policy-based barriers, such as inequitable advancement opportunities and gendered role expectations (i.e., family-friendly workplace), while the societal level encompasses broader cultural and normative influences, including stereotypes and historical biases about women in sport (i.e., ideology of male superiority in sport). The socioecological model was not used as a coding frame, but rather as an interpretive lens during analysis and discussion, helping to situate the inductively generated themes within multilevel systems of influence. This approach reflects the reality that participants' experiences often traversed multiple levels simultaneously, illustrating the complexity of gendered dynamics in professional men's sport.

Thematic analysis followed Braun and Clark's (37) six-phase framework. First, the primary author immersed herself in the data by listening to audio recordings and repeatedly reading transcripts to gain a deep familiarity with participants' accounts. Using NVivo (Version 14), transcripts were then coded systematically to capture features relevant to the research questions. Codes were reviewed and grouped into preliminary themes reflecting conceptual similarities across the dataset. These themes were iteratively refined and checked against the data to ensure coherence and distinction. Final themes were defined, named, and structured to capture the essence of participants' experiences, acknowledging the interplay of individual, interpersonal, organizational, and societal influences. The resulting themes were then integrated into the findings and discussion, supported by illustrative quotations and interpretive analysis.

## 3 Results

Four primary themes were identified, reflecting the personal and professional challenges faced by participants, as well as the supports and barriers influencing their careers: (1) Establishing professional identity in a male-dominated environment, (2) Navigating sexual objectification, (3) Career progression and organizational barriers, and 4) Work-life balance and personal costs. Collectively, these themes provide a nuanced understanding of the multi-level factors shaping female physiotherapists' experiences in a male-dominated professional sport context, with the findings presented below.

### 3.1 Establishing professional identity in a male-dominated environment

Participants described the challenges of establishing professional identity in a male-dominated environment, particularly when in junior roles. Many struggled to gain trust and recognition from male colleagues and players. “As a junior physiotherapist, I'm struggling a lot with my colleagues to gain their trust” (PT 8). Opportunities for open discussion or professional debate were rare, leaving some participants feeling excluded from decision-making, “It'd be nice to sometimes have debates [around injury management] and to feel that my opinion is valued” (PT 3). Hierarchical and gendered power relations compounded this sense of marginalization. One participant described feeling silenced in her interactions with a senior male colleague, “I found him really intimidating.. I didn't feel like I could even say hello to him” (PT 11). Being both young and the only female compounded feelings of isolation and powerlessness, “At first, I also struggled a little bit with them because of trust issues, so they would ask for a second opinion from my other colleagues or choose my colleagues to go for treatment over me” (PT 8). These dynamics made it difficult for junior physiotherapists to assert themselves or have their professional opinions valued. This was also a common reflection for participants who had managed sustained employment in professional rugby. PT 1 remembered, “ [everything] was totally new, and I didn't have rugby experience. I wouldn't be the most confident person. So I think my personality probably would have made people a bit more hesitant as well…it was quite a tough environment”. Professional identity often developed with experience,

As you get older and you realize where the professional boundary lies and how important your professionalism is for future physios coming through, you've got to be clever and think about what you do and how you want to portray yourself as a female physio in this industry. (PT 12).

On other occasions the participants changed their approach to not “bruise the ego” of male colleagues,

I think as I've gotten older and, in the profession, more, I've learned sometimes that even if I know the answer for things, it's not necessary to give the answer. It's sometimes just to ask the question that will lead to the idea that I had. (PT 5).

### 3.2 Navigating sexual objectification

Participants consistently reported feeling held to stricter professional standards than male colleagues, requiring constant self-monitoring in dress, behavior, and social interactions,

I think as a female; I've often had to be more professional than male staff in the environment that I'm in just because I'm working with male players… I've had to be very strict on my boundaries and who I am and what I am. (PT 5).

Professional boundaries extended into social contexts, with PT 4 noting, “You do feel like those nights out are really fun, but you are still working. I had to be so careful… I don't think the male physio's would have that pressure; they were just going out with the team”. Participants also described additional scrutiny regarding their appearance and behavior, “I’ll see my male colleagues joke around with the team, connect on social media, and meet up for drinks. Whereas I don't feel I could do that, rightly or wrongly,” (PT 12) and, “You need to present yourself a little bit differently and maybe be more sensitive to the work environment.. how you dress, how you present yourself around the club, how you go on away trips, what you do on away trips, knowing where you have to stand” (PT 5). The age and experience level of the physiotherapist were also influential here, with PT 4 commenting “I should have called them out [on inappropriate comments], but I didn't because I was the most junior in that organisation. I wasn't brave enough, if I'm honest”

Gender stereotypes and bias further shaped professional identity. Participants described contradictory expectations around femininity and professionalism. “You've got to look reasonably thin and fit, but you can't be too good looking, because then you're in trouble…you need to be a mum or a sister, but you can't be too mumsy, because then you're too soft” (P12). Several participants reported sexual objectification and inappropriate comments that went unaddressed, “Sometimes I received sexual comments by coaches and players in the team throughout the three years I worked there, which went unaddressed by the organization” (PT 2). Such experiences reinforced a culture of silence.

I think as a female in a male environment we have spent too long being silent and enduring male behavior…Making sexual comments, or innuendos, or jokes then having to absorb that behavior silently and not be allowed to respond…If we do respond, it's almost like I have created the offence and it's my fault. (PT 9).

Being the only female on the team compounded these feelings:

It was hard to bring up issues in the environment because I felt like I was not in the position to do so. I was the youngest physiotherapist and the only female. It was my dream as a physio to work there, so I wasn't in the position to do anything about it. (PT 2)

### 3.3 Career progression and organizational barriers

Participants reported persistent difficulties advancing to senior roles due to gendered biases, often feeling overlooked despite their qualifications. PT 10 stated, “They will find a reason not to hire you as a girl, and it won't be related to your qualifications,” while another explained, “It did take me much longer to get into the senior structure than any other male…I always say that in this environment, it takes females around six years to achieve what a male does in one” (PT 9). Being one of the only females in rugby union further compounded the challenge, “In rugby especially, I think it's quite hard for a female…I think there is another female physiotherapist…with another team, but I have never seen others apart from her. I think they prefer males for the roles” (PT 11).

Exclusion from opportunities was often reinforced by informal networks. PT 7 described,

I asked for the opportunity to backfill the [competition] role, but I was advised I wouldn't be considered because I'm female, and they were looking for a male. They said, we'll be looking for somebody who can be on the pitch and hold tackle bags, and…that wouldn't be a female doing that. (PT 7).

Participants also reflected on the influence of the “old boys’ network,” with PT 10 noting:

You only get hired by who you know, like so boys tap each other on the shoulder…I interview well, my resume is really good, but then someone's always going to pick a guy that technically has more experience in some way. It'll be called experience. (PT 10)

Senior female physiotherapists further highlighted underrepresentation in leadership: “What I'm now finding as a challenge is that the numbers of females [in leadership positions]… there are minimal to none. I'm the only female in the senior leadership group, which I have found really challenging” (PT 7).

The absence of female role models emerged as a significant challenge. With support often coming from male colleagues, “There really aren't that many females that I can look up to” (PT 3) and “as a role model, it's been more my male colleagues from previous roles” (PT 7). Competition and insecurity among female colleagues hindered supportive relationships,

I do find female physios don't work so well together in a professional environment. I think they're very competitive with each other. If you come from a place where you're insecure about your own skill, you feel like you need to prove yourself the whole time. (PT 5)

Structural and organizational challenges extended beyond career progression. Participants highlighted low remuneration, often needing other sources of income “I organized to work the away games in Autumn instead of later in the season so that I can do my [other job]. My salary was not high enough, so I needed to add another job” (PT 11). Financial pressures disproportionately affected those with family responsibilities, with PT 4 noting, “I was on such a low wage for the hours.. as soon as you put nursery fees into that, my salary didn't cover nursery fees. It's crazy. So, I was paying to go to work.” Gender pay disparities were also highlighted: “I know the physio before me in my role, and he got paid way more than I've been paid” (PT 9).

The lack of organizational infrastructure, including HR support, facilities, and travel arrangements, exacerbated these challenges. PT 10 described:

There was no support system whatsoever. I tried to do what I could to improve the department but that didn't get noticed. There was no formal communication or feedback loop… They performed tick box exercises but there was no meaningful follow-through. (PT 10)

Inequities in leave and support were evident, “One of the guys in the [role] was burnt out in the first year I was there, and they gave him six months off with [support]… whereas I got two weeks off and then basically got told to leave” (PT 10). Facilities for female staff were also often inadequate, “There aren't female changing rooms or separate toilets in some of the stadiums and training grounds” (PT 2). Team uniforms were also an issue. PT 10 reported, “you never feel part of a team when you don't get ordered clothes to fit you because it was an extra hassle”. While, another participant highlighted barriers that came with being the only female and travelling with the team:

Yeah, I do travel and sometimes this is a problem. If there are two physiotherapists flying with the team, if they are both male, they can share a room. But if I travel, they have to organise two rooms. So, for example, when the team goes to [country], they stay there for two weeks and so of course I want to go. But they will take two males because of financial reasons. (PT 11)

This was further emphasised by, “I was not allowed to travel some of the away trips… because I'm female… if you're not married then they're like sorry we don't want a girl travelling with that’s not married” (PT 5).

### 3.4 Work-life balance and personal costs

The intensity of the work, long hours, and extensive travel often interfered with personal relationships and family planning. PT 1 explained, “I don't really have much of a personal life. It's very long hours and I really struggle to find that balance between work and personal life”, while PT 5 reflected, “I'm still not married.. and I've been working 10 years in rugby, so I don't know if that tells you. I’m married to rugby.” She continued,

I don't know if I'd be in rugby if I had a family. I think the responsibilities and the time away from home.. once I get married and have children, I don't think the environment, especially for females, allows it. (PT 5).

Participants reported that these demands often resulted in emotional exhaustion, “It's such a personable job where you've got to be, go, go, go all the time, it's very fast-paced. By the time you get home, you're so shattered that you can't really bother to do anything” (PT 12). Travel schedules and irregular hours also affected relationships, with PT 5 noting, “I think the fact that it's often a 6–7-day week makes it difficult”. This was reiterated by PT 7 who explained how she had to miss out on important personal engagements due to unsocial work hours, travel commitments, and prioritising the players over her personal time:

It has impacted [me personally] in the sense of the number of hours and the demands of the role mean that at times either you don't have the energy or the time to socialise with friends or family. There have definitely been certain things in the past that I've maybe missed out on. (PT 7)

These pressures extended to balancing family life and other personal responsibilities, highlighting the personal sacrifices required to remain in professional rugby environments. Having supportive partners allowed some participants to navigate the demands of their profession while maintaining family responsibilities in that “I've got a very, very supportive husband. And I always say if it wasn't for him, I mean, who would allow their wife to travel with 37 other males? There are not many husbands that would do that” (PT 9). Participant 4 reiterated how her partner had to accommodate the demands of her career, explaining that, “my husband has to reduce his work hours.. so planning ahead is key when it comes to the traveling component” (PT 4).

## 4 Discussion

This study explored the barriers and supports experienced by female physiotherapists working in professional men's rugby union. The findings highlight the entangled nature of these experiences, which do not sit neatly within discrete ecological levels, but instead traverse individual, interpersonal, organizational and societal domains, supporting the systemic paradigm noted in Bronfenbrenner's (38) original ecological model. This complexity reflects the challenges of navigating male-dominated environments, where gendered expectations, structural inequities, and professional hierarchies intersect. While participants acknowledged the fulfilling, dynamic aspects of their work, their experiences highlighted persistent inequities, gendered double standards, and structural barriers that continue to shape opportunities for career progression. These findings resonate with previous research on women in male-dominated professions (39), while offering further insights into the intersection of gender, professional practice, and organizational culture in professional men's rugby. Specifically, there are significant changes in how female physiotherapists are viewed based on their age, with older physiotherapists viewed as mother figures and younger ones deemed to be there “just to find a husband”. Similarly, while policy and open communication practices are aimed at gender equity and supporting the acceptance of women in sport, there is still an historical undertone of accepted practices that allows sexism to go unchallenged.

Participants in this study frequently reported experiences of gender bias, stereotyping, and microaggressions. These ranged from being underestimated due to their physical size and questioned about their rugby knowledge, to being viewed as potential romantic partners for players. Like much of the sport industry, these experiences are more frequent for younger women (3). These experiences align with prior research that highlights how gender stereotypes undermine women's authority and progression in male-dominated sports (21, 39) Many participants reported adopting strategies such as developing a “thick skin” or modifying their behavior to “fit in”. While these attributes facilitated survival, they also reflect the burden placed on women to adapt to environments that remain rooted in masculine norms (40). The normalization of such behavior risks perpetuating cultures of exclusion. Future interventions must aim to disrupt these dynamics, ensuring that microaggressions and bias are addressed directly rather than managed individually by women.

A significant lack of organizational support, with participants describing exclusion from clinical decision-making, lack of maternity policies, and inadequate responses to conflict and discrimination, was evidenced in the findings. These structural barriers mirror wider evidence that sporting organizations often advocate gender equality yet fail to implement meaningful action (41). Several participants also perceived bias in hiring and promotion practices, with less experienced male colleagues advancing ahead of them. Younger physiotherapists also missed out on development opportunities by not being allowed to travel with the team. This resonates with longstanding critiques of “old boys’ networks” in sport, where leadership remains overwhelmingly male (39). Addressing these inequities requires structural reform, including transparent hiring and promotion processes, robust grievance reporting mechanisms, and inclusive workplace policies such as maternity leave and childcare support. Such systemic measures are crucial not only for retention but also for enabling progression into senior physiotherapy and leadership roles. Without organizational change, women remain at risk of departure from the male-dominated workforce, reinforcing gender imbalances.

A lack of visible female role models was a barrier for participants, echoing findings across coaching, sports medicine, and leadership (13). Participants noted how the absence of women in senior roles undermined their sense of belonging and limited their aspirations for progression. Visibility extends beyond numbers; recognition of women's contributions within teams is also essential. Participants described frustration at being excluded from decision-making or having their input disregarded, experiences that diminish professional credibility and reinforce stereotypes of women as less authoritative. Improving visibility requires organizations to actively promote women into positions of responsibility, highlighting their expertise and achievements. Gender quotas may be required to address this, not as tokenism, but as a tool to reset imbalances and shift culture (42). Celebrating female physiotherapists' career stories as role models, not exceptions, inspires others and helps normalize women's leadership in rugby (1). Physiotherapy studies at university level should integrate more opportunities for female students to gain exposure to elite sports settings through targeted internships, shadow programs, and leadership training (43). Increasing the representation of female physiotherapists in professional rugby not only provides role models for younger practitioners but also challenges entrenched cultural perceptions about women's competence in male-dominated sport.

Supportive relationships with colleagues, players, and mentors were among the strongest facilitators reported. Participants described positive camaraderie with athletes, who often valued their empathy and listening skills, challenging assumptions that male athletes resist working with female practitioners. Relationships with colleagues were also important, with male physiotherapists often providing mentorship and guidance. However, reliance on male mentors presents limitations, as their perspectives may not fully reflect the unique challenges women face in male-dominated settings. Previous research suggests that female mentorship can foster belonging, confidence, and resilience (44, 45). Yet, participants also described tensions with female colleagues, reflecting the competitive pressures of underrepresentation in male-dominated environments (18). Building formal mentorship networks, both within rugby and across sports, may provide female physiotherapists with crucial support systems, while reducing reliance on informal or male-dominated networks.

The personal costs of sustaining a career in professional men's rugby, including long hours, travel demands, disrupted social and family life, and emotional exhaustion, were significant for participants. These challenges are not merely individual burdens but are rooted in organizational practices that fail to adequately support work-life integration (2, 14). Framing work-life balance as an organizational responsibility underscores the need for structural interventions, including scheduling flexibility, travel support, equitable leave policies, and family-friendly provisions (45). Organizational commitment to these measures is critical to reducing departure from the profession, promoting retention, and ensuring that female physiotherapists can thrive professionally without disproportionate personal sacrifice (46). By recognizing and addressing these structural responsibilities, professional environments can shift the onus from individual coping to systemic accountability, creating sustainable careers for women working in professional sport.

This study, while providing valuable insights, has a few limitations that may impact the interpretation and generalizability of the findings. First, the small sample size may limit the generalizability of the results across the wider population of female physiotherapists working elsewhere in professional men's rugby union. Additionally, as the subject matter involved sensitive topics relating to gender and professional experiences, some participants may have been hesitant to share certain details openly and may have been concerned about potential identification, even with assurances of confidentiality and anonymity. This may have led to self-censorship or withholding of experiences that could have offered additional depth. The study did not explore the gender identities that the female participants may have developed or assigned themselves; nor were other determinants such as race and sexual orientation used as a consideration in analysis. This does create a limitation in the findings, suggesting that there was a homogeneity among the group which may have been an incorrect assumption. Further background information aligned to these factors may have revealed nuances in the experiences of the group which this analysis has overlooked. The findings of this study are also context-specific to men's rugby union and therefore may limit its applicability to other male-dominated sports where cultural and organizational differences may yield different experiences for female physiotherapists. Future research should also examine the embodied and practice-based aspects of physiotherapy in elite collision sports, with greater attention to gendered experiences. Research is needed on perceptions of physical competence in manual therapy, injury management, and high-contact environments such as rugby. This would provide a more comprehensive understanding of how gender shapes the physical and interpersonal demands of physiotherapy practice.

## 5 Perspectives

To tackle microaggressions effectively mandatory training for everyone working in professional sport on unconscious bias and inclusive behaviors is needed. But training alone, is not enough. Real-time accountability through clear feedback systems must be created, so when lines are crossed, people are held responsible immediately. Others should speak up, without fear of consequence, as silence only allows bias to persist. A “red flag’ system can be implemented to better track patterns of behavior and allow systemic issues to be addressed. Gender equity policies must move beyond words on paper to real action, with measurable goals and transparency. Family-friendly policies and flexible work options, including strong maternity return support, should be standard to support work-life balance for everyone. Facilities and resources should be accessible and feasible to all. This means everything from uniforms to changing rooms and travel accommodations tailored to everyone's needs. Leadership development is crucial; mentorship and upskilling for female physiotherapists opens pathways for career advancement. Finally, promotion and hiring processes must be transparent, and ongoing research should track progress to hold governing bodies accountable.

## 6 Conclusion

This study examined the experiences of female physiotherapists working in professional men's rugby union, revealing how gendered expectations, organizational barriers, and cultural norms intersect to shape their professional identities and career trajectories. Despite deriving satisfaction and pride from their roles, participants described persistent inequities, microaggressions, and exclusion from decision-making, often requiring them to self-monitor behavior and adapt to masculine workplace cultures. The findings highlight the need for structural and cultural reform across multiple levels, including transparent promotion pathways, inclusive family leave policies, active mentorship networks, and accountability for gender bias and microaggressions. Enhancing visibility and representation of female physiotherapists within elite sport is also essential to challenge stereotypes and foster belonging. Achieving genuine equity will require not only policy change but also sustained cultural transformation, creating professional environments where women's expertise is recognized, respected, and equitably supported.

## Data Availability

The data are not publicly available due to their containing information that could compromise the confidentiality of research participants. Requests to access the datasets should be directed to Fraser Carson, fcarson@lunex.lu.

## Footnotes

^1^The authors recognize that sex and gender have been used interchangeably in the literature and gender has become more commonly reported within gender equity studies. Throughout the main text, we use 'women' as a noun and 'female' as an adjective. Within the results we have kept specific wording of the participants.
